# Changes in fatigue, autonomic functions, and blood biomarkers due to sitting isometric yoga in patients with chronic fatigue syndrome

**DOI:** 10.1186/s13030-018-0123-2

**Published:** 2018-04-10

**Authors:** Takakazu Oka, Tokusei Tanahashi, Nobuyuki Sudo, Battuvshin Lkhagvasuren, Yu Yamada

**Affiliations:** 1 Department of Psychosomatic Medicine, International University of Health and Welfare hospital, Iguchi 537-3, Nasushiobara-shi, Tochigi-ken 329-2763 Japan; 2 Department of Psychosomatic Medicine, Graduate School of Medical Sciences, Kyushu University, Fukuoka, 812-8582 Japan

**Keywords:** Chronic fatigue syndrome, Isometric yoga, Myalgic encephalomyelitis, Cytokine, Heart rate variability, DHEA-S, Treatment

## Abstract

**Background:**

In a previous randomized controlled trial, we found that sitting isometric yoga improves fatigue in patients with chronic fatigue syndrome (CFS) who are resistant to conventional therapy. The aim of this study was to investigate possible mechanisms behind this finding, focusing on the short-term fatigue-relieving effect, by comparing autonomic nervous function and blood biomarkers before and after a session of isometric yoga.

**Methods:**

Fifteen patients with CFS who remained symptomatic despite at least 6 months of conventional therapy practiced sitting isometric yoga (biweekly 20 min practice with a yoga instructor and daily home practice) for eight weeks. Acute effects of sitting isometric yoga on fatigue, autonomic function, and blood biomarkers were investigated after the final session with an instructor. The effect of a single session of sitting isometric yoga on fatigue was assessed by the Profile of Mood Status (POMS) questionnaire immediately before and after the session. Autonomic nervous function (heart rate (HR) variability) and blood biomarkers (cortisol, DHEA-S, TNF-α, IL-6, IFN-γ, IFN-α, prolactin, carnitine, TGF-β1, BDNF, MHPG, and HVA) were compared before and after the session.

**Results:**

Sitting isometric yoga significantly reduced the POMS fatigue score (*p* < 0.01) and increased the vigor score (*p* < 0.01). It also reduced HR (*p* < 0.05) and increased the high frequency power (*p* < 0.05) of HR variability. Sitting isometric yoga increased serum levels of DHEA-S (*p* < 0.05), reduced levels of cortisol (*p* < 0.05) and TNF-α (*p* < 0.05), and had a tendency to reduce serum levels of prolactin (*p* < 0.1). Decreases in fatigue scores correlated with changes in plasma levels of TGF-β1 and BDNF. In contrast, increased vigor positively correlated with HVA.

**Conclusions:**

A single session of sitting isometric yoga reduced fatigue and increased vigor in patients with CFS. Yoga also increased vagal nerve function and changed blood biomarkers in a pattern that suggested anti-stress and anti-inflammatory effects. These changes appear to be related to the short-term fatigue-relieving effect of sitting isometric yoga in patients with CFS. Furthermore, dopaminergic nervous system activation might account for sitting isometric yoga-induced increases in energy in this patient population.

**Trial registration:**

University Hospital Medical Information Network (UMIN CTR) UMIN000009646. Registered Dec 27, 2012.

## Background

Chronic fatigue syndrome (CFS) is a debilitating disease characterized by persistent or relapsing unexplained fatigue of at least six month of duration that is not relieved by rest and causes a substantial reduction in daily activities [1].

In a previous randomized controlled trial, we demonstrated that sitting isometric yoga improved fatigue and pain in patients with CFS who were resistant to conventional therapy [2]. In this trial, the Chalder fatigue score in patients who, in addition to receiving conventional pharmacotherapy, practiced 20-min of sitting isometric yoga for two months was significantly lower than in patients who received conventional therapy alone. This finding suggests that regular practice of sitting isometric yoga reduces the severity of fatigue. Furthermore, the Profile of Mood States (POMS) fatigue (F) score decreased and the POMS vigor (V) score increased after a single yoga session. These scores were measured before and after the final session with a yoga instructor, suggesting that even a 20-min sitting isometric yoga session can reduce fatigue and increase energy in patients with CFS when they are accustomed to the procedures and can practice it successfully.

It remains unclear how sitting isometric yoga reduces fatigue and increases energy in patients with CFS. Previous studies have suggested several abnormalities in patients with CFS, including dysfunctions of the autonomic nervous system (ANS), the hypothalamic-pituitary-adrenocortical (HPA) axis, the immune system, and the central nervous system (CNS) [3]. Autonomic dysfunction is characterized by low vagal tone and sympathetic overactivity [4, 5]. For example, in patients with CFS, baseline heart rate (HR) is higher than in healthy subjects [4–6]. Dysfunctions of the HPA axis in patients with CFS include attenuated diurnal changes in cortisol [7, 8] and decreased dehydroepiandrosterone sulfate (DHEA-S) [9–11]. Dysfunctions of the immune system include increased blood levels of multiple cytokines, including transforming growth factor (TGF)-β1 and several proinflammatory cytokines such as tumor necrosis factor (TNF)-α, interleukin (IL)-6, and interferon (IFN)-γ [12–15]. Dysfunctions of the CNS include hypoactivity of the dopaminergic nervous system to reward stimuli [16, 17]. Other dysfunctions in patients with CFS include lower blood levels of acylcarnitine than in healthy subjects [18, 19].

To date, it is not known if sitting isometric yoga can improve these abnormalities in patients with CFS. Previous studies have demonstrated that yoga counteracts some of the above abnormalities in other clinical populations. For example, yoga is known to change autonomic function from a sympathetic nerve-dominant state to a parasympathetic nerve-dominant state in healthy subjects [20]. Yoga has also been reported to improve dysfunctions of the HPA axis, e.g. the blunted cortisol diurnal slope [21] and to reduce peripheral pro-inflammatory markers such as nuclear factor kappa B (NF-κB) and TNF-α [22, 23] in cancer patients. In accordance with these findings, yoga has been shown to reduce fatigue and increase energy in current cancer patients and in survivors [21, 24]. Therefore, we hypothesized that yoga improves some of these dysfunctions in patients with CFS, which results in the fatigue-relieving effect of yoga.

We also hypothesized that, in patients with CFS, the short- and long-term fatigue-relieving effects of yoga might be caused by different mechanisms, based on our observations of patients who regularly practice yoga. That is, the short-term effect, which is observed within 30 min of practicing yoga, is associated with changes in autonomic and neurotransmitter functions within the brain and with reduced levels of stress hormones such as cortisol. On the other hand, the long-term effect, which is gradually obtained over eight weeks, is associated with changes in immune function, nutritional state, and behavioral (coping) styles. We were particularly interested in the mechanisms underlying the short-term fatigue-relieving effect and focused on this analysis in the present study.

To elucidate the mechanisms underlying the acute, fatigue-relieving effect of sitting isometric yoga in patients with CFS, we measured changes in autonomic functions and blood biomarker levels before and immediately after a yoga session and assessed the correlation between these parameters and yoga-induced fatigue reduction and increased vigor.

## Methods

### Subjects

The study included 15 patients with CFS who were enrolled in the yoga group of our previous randomized, controlled trial [2]. All study participants were outpatients with CFS who visited the Department of Psychosomatic Medicine of Kyushu University Hospital and met the following inclusion criteria: (1) the subject’s fatigue had not significantly improved despite at least six months of conventional treatment [25–27]; (2) the subject was between 20 and 70 years old; (3) the subject’s level of fatigue was serious enough to cause an absence from school or the workplace for at least several days a month but not serious enough to require assistance with the activities of daily living; (4) the subject was able to fill out questionnaires without assistance; (5) the subject could sit for at least 30 min; and (6) the subject could visit the hospital every two or three weeks. The diagnosis of CFS was made based on diagnostic criteria of the 1994 international research case definition of CFS [1]. Patients also met the 2011 international consensus criteria for myalgic encephalomyelitis [28] and the 2015 diagnostic criteria for systemic exertion intolerance disease [29].

### Methods

**Fig. 1:**
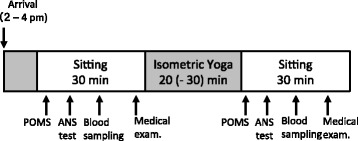
Experimental procedures

Participants practiced sitting isometric yoga regularly for about eight weeks. That is, patients were fully accustomed with the procedures of the sitting isometric yoga program prior to evaluation. Assessments were made at the final yoga session, which was done with an instructor at the hospital between 2 pm and 4 pm. Patients first filled out the POMS self-rating questionnaire and underwent non-invasive autonomic function testing and blood sampling. Patients then practiced sitting isometric yoga in a quiet room for 20 min (sometimes up to 30 min) with an experienced yoga instructor (guided yoga session). After the yoga session, the patient again filled out the POMS questionnaire, underwent autonomic function testing and blood sampling, and had a medical check-up by his/her physician (Fig. 1). The POMS questionnaire was collected by a nurse who was not involved in the study. Blood samples were centrifuged and stored at − 80 °C until measurements were performed.

### Yoga intervention

**Fig. 2:**
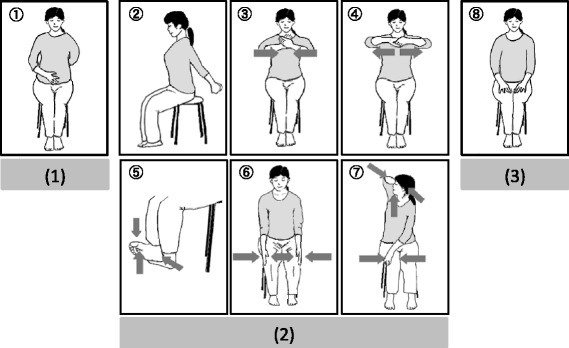
Sitting isometric yoga program. The 20-min sitting isometric yoga program consisted of three parts. (1) Patients practiced being aware of their spontaneous breathing for 1 min to facilitate interoceptive and proprioceptive awareness. (2) Patients slowly practiced six isometric postures, taking 3–10 s each (5 s in average), in association with exhalation with/without sounds, using 50% maximal physical strength. (3) Patients practiced abdominal breathing for 1 min

We developed a 20-min sitting isometric yoga program for patients with CFS so as not to induce post-exertional malaise [2] and adverse events [30, 31]. All participants practiced sitting isometric yoga, consisting of biweekly, 20-min sessions with a yoga instructor and daily in-home sessions, for about eight weeks. On the experimental day, patients practiced sitting isometric yoga in a quiet room on a one-to-one basis with an instructor who has over 30 years of experience (Fig. 2). The precise procedures of sitting isometric yoga have been described elsewhere [2]. In brief, the program consisted of three parts. First, patients practiced being aware of their spontaneous breathing with the right hand on the lower abdomen and the left hand on the back for one minute to facilitate interoceptive and proprioceptive awareness. Second, patients practiced 6 isometric poses 4–6 times, 2–3 times with sounds like “Uh…” or “Ah….” followed by 2–3 times without sounds. Isometric postures were practiced slowly, taking 3–10 s (5 s in average) in association with exhalation, using 50% of maximal physical strength. Tension was then slowly released with inhalation. Finally, the hands and body returned slowly to the basic, sitting position taking 3–10 s (5 s in average) with exhalation with/without sounds. The length and the number of repetitions varied depending on the patient’s severity of fatigue and psychological and physiological tension. Third, the patients observed abdominal breathing for one minute. Patients were not asked to practice abdominal breathing intentionally, rather, if they practiced sitting isometric yoga successfully, their breathing pattern became more regular, slower, deeper, and abdominal when compared to the breathing before practicing yoga. Patients were asked to observe such breathing.

### Monitoring of yoga practice

Patients were monitored while practicing sitting isometric yoga in two ways: by a yoga instructor and through a “yoga diary”. When the patients visited the hospital, they practiced sitting isometric yoga in a quiet room for 20 min on a one-to-one basis with an instructor who monitored and analyzed their practice, and made changes if necessary. Most patients visited their doctors every two or three weeks during the intervention period. Therefore, all patients practiced this yoga program at least four times (5.6 times, on average) with the instructor during the intervention period. In addition to receiving a private lesson, the patients were asked to practice the yoga program on non-class days if they could, with the aid of a digital videodisc (DVD) and a booklet. The patients were also asked to keep a “yoga diary” in which they recorded the amount of time they practiced and any questions they had during the practice. On the day of the visit, the yoga instructor and the patient’s doctors checked the diary. According to the diaries, they practiced sitting isometric yoga at home for a mean of 5.7 times/week during the intervention period.

### Measures

#### Fatigue and vigor

To assess the acute effects of sitting isometric yoga on fatigue and vigor, the F and V scores of the POMS [32] self-rating questionnaire were assessed immediately before and after a guided yoga session.

#### ANS function

Non-invasive ANS function testing was conducted by analyzing heart rate variability (HRV). After a sufficient resting period followed by time to fill out the POMS questionnaire, patients underwent a 3-lead electrocardiogram (ECG) of 2 min duration while in the sitting position. Electrodes were placed on both wrists. Beat-to-beat HR was assessed on the ECG, and HRV indices and respiratory rate were measured using the “Kiritsu Meijin” (Crosswell Co., Inc., Yokohama, Japan) [33], which included a HR monitor LRR-03 (GMS, Tokyo, Japan). The majority of previous studies have used spectral techniques based on the Fast Fourier Transform (FFT). However, FFT is insufficient to estimate the precise power spectral density from short time series data. The MemCalc method [34] is a new technique for time series analysis. It is a combination of the maximum entropy method for spectral analysis and the non-linear least squares method for fitting analysis. This enabled us to achieve a reliable analysis of the low- (LF; 0.05–0.15 Hz) and high-frequency components (HF; 0.15–0.4 Hz) over a minimum interval of 30 s. Time domain analysis and spectral analyses of HR variability using the MemCalc system were performed over a 1-min period. In the time domain analysis, the coefficient of variation of R-R intervals (CV_R-R_) = component coefficient of variance (total power) was shown. HF was used as an index of parasympathetic activity, while LF was used as a mixed index of sympathetic and parasympathetic activity. LF/ HF was used as an index of sympathetic activity [34–36].

### Blood biomarkers

#### Serum markers

Serum levels of DHEA-S were measured using a chemiluminescent enzyme immunoassay (CLEIA). Cortisol and prolactin (PRL) were measured by electro-chemiluminescence immunoassay. Total carnitine, acylcarnitine, and free carnitine were measured by the enzyme cycling method. IL-6 was measured using a human IL-6 CLEIA cartridge (Fujirebio, Tokyo, Japan), with a minimum detectable concentration of 0.2 pg/mL. TNF-α was measured by enzyme-linked immunosorbent assay (ELISA) using Quantikine high-sensitivity ELISA human TNF-α immunoassay (R&D Systems, Inc., Minneapolis, MN, USA), with a minimum detectable concentration of 0.07 pg/mL. IFN-α was measured using the VeriKine™ Human Interferon Alpha Multi-Subtype Serum ELISA Kit (pbl assay science, Piscataway, NJ, USA), with a minimum detectable concentration of 12.5 pg/mL. IFN-γ was measured by enzyme amplified sensitivity immunoassay (EASIA) using the MEDGENIX human IFN-γ EASIA kit (BioSource Europe S.A., Nivelles, Belgium), with a minimum detectable concentration of 0.1 IU/mL.

#### Plasma makers

Plasma levels of 3-methoxy-4-hydroxyphenylglycol (MHPG) and homovanillic acid (HVA) were measured by high performance liquid chromatography. Brain-derived neurotrophic factor (BDNF) was measured using the Quantikine ELISA human BDNF kit (R&D Systems, Inc., Minneapolis, MN, USA), with a minimum detectable concentration of 20 pg/mL. TGF-β1 was assessed using the Quantikine ELISA human TGF-β1 kit (R&D Systems, Inc., Minneapolis, MN, USA), with a minimum detectable concentration of 0.50 pg/mL.

### Statistical analyses

The normality of data distribution was evaluated by the Kolmogorov-Smirnov test. Differences in POMS scores, plasma/serum components, and physical parameters before and after the yoga session were analyzed using a paired-sample *t* test or the Wilcoxon signed-rank test if the Kolmogorov-Smirnov test was significant. Correlations between POMS scores and plasma/serum components and physical parameters were identified using bivariate Pearson’s test. Correlated plasma/serum components and physical parameters (as independent variables) were further analyzed using multiple linear regression analysis to explain the causal relationships with the respective POMS scores (as dependent variables) using pairwise deletion for missing values. To determine any difference in the POMS fatigue and vigor scores with a two-sided significance level of 0.05 and with at least 80% power, an appropriate sample size of 15 was calculated using the mean and standard deviation between two groups with different POMS scores reported in a previous study [37]. A post-hoc power analysis using the G*Power test computed that the achieved power for the paired-sample, two-tailed *t* test in 15 subjects was 99%. Statistical significance was set at *p* < 0.05 and all tests were two-tailed. Data are presented as means ± standard deviations (SD) and 95% confidence intervals (95% CI). Data were analyzed using SPSS for Windows, V.21.

## Results

This study included 15 subjects who completed the yoga intervention in a previous study (age range: 24–60 years; mean age (mean ± SD): 38.0 ± 11.1 years; 3 men) [2]. One patient failed to undergo ANS function testing. One patient failed to give a blood sample for MHPG and BDNF testing, and two patients failed to give samples for TGF-β1, DHEA-S, and cortisol testing. The missing data were excluded from the analysis. No patient was treated with hydrocortisone.

### Effect of sitting isometric yoga on fatigue and vigor

The effects of sitting yoga on fatigue and vigor were assessed by comparing the POMS F and V scores before and after the final guided yoga session. After the session, the mean F score decreased significantly (from 21.9 ± 7.7 to 13.8 ± 6.7, *P* = 0.001) and mean V score increased significantly (from 17.8 ± 7.6 to 22.9 ± 8.2, *P* = 0.002; Table 1). These values were reproductions from our previous study [2].

**Table 1:**
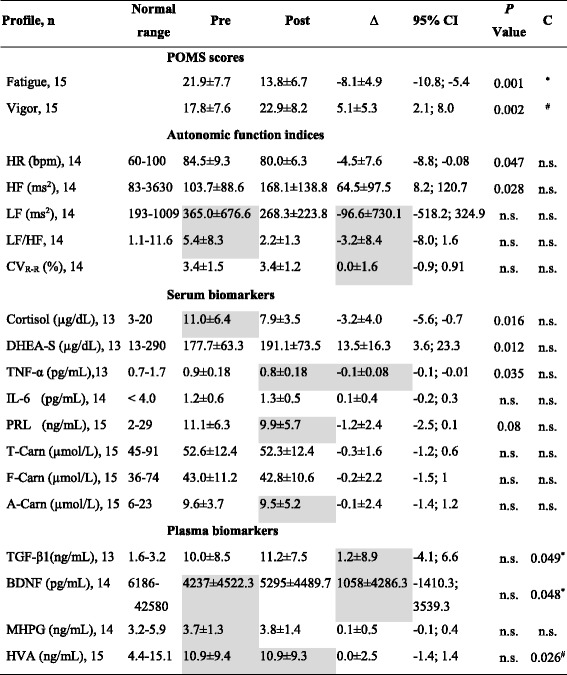
Changes in POMS scores, autonomic function indices, and blood biomarkers following a sitting isometric yoga session

n: Sample size

Values are mean ± standard deviation

Pre: Mean values before yoga sessions

Post: Mean values after yoga sessions

∆: The mean difference between Pre and Post values, i.e. Post value – Pre value

*P* value: Paired *t*-test or Wilcoxon signed-rank test between Pre and Post values

95% CI: 95% Confidence interval (Lower bound – Upper bound)

C: Correlations

*: Correlation between ∆ F and ∆ plasma/serum components, *P* < 0.05

#: Correlation between ∆ V and ∆ plasma/serum components, *P* < 0.05

n.s.: not significant

Highlighted in gray: The probability of normal distribution is violated by Kolmogorov-Smirnov test

### Effects of sitting isometric yoga on ANS functions

The effects of sitting isometric yoga on ANS function were assessed by comparing the HR, CV_R-R_, HF- and LF- components of the HRV and LF/HF before and after yoga. After the guided yoga session, HR decreased significantly (from 84.5 ± 9.3 bpm to 80.0 ± 6.3 bpm, *P* = 0.047) and HF increased significantly (from 103.7 ± 88.6 ms^2^ to 168.1 ± 138.8 ms^2^, *P* = 0.028), whereas CV_R-R_, LF and LF/HF did not significantly change (Table 1). Respiratory rate both before and after the yoga session was 17 /min.

### Effects of sitting isometric yoga on blood biomarker levels

To elucidate possible mechanisms behind the acute fatigue-relieving effect of sitting isometric yoga, we compared the blood levels of several biomarkers before and after the guided yoga session. These biomarkers included serum cortisol, DHEA-S, TNF-α, IL-6, IFN-α, IFN-γ, PRL, total carnitine, free carnitine, and acylcarnitine, and plasma TGF-β1, BDNF, MHPG, and HVA (Table 1).

We found that DHEA-S significantly increased (from 177.7 ± 63.3 μg/dL to 191.1 ± 73.5 μg/dL, *P* = 0.012), whereas cortisol and TNF-α significantly decreased (from 11.0 ± 6.4 μg/dL to 7.9 ± 3.5 μg/dL, *P* = 0.016 and 0.9 ± 0.18 pg/mL to 0.8 ± 0.18 pg/mL, *P* = 0.035, respectively) and PRL had a tendency to decrease (from 11.1 ± 6.3 ng/mL to 9.9 ± 5.7 ng/mL, *P* = 0.077) after the yoga session. Serum levels of total carnitine, free carnitine, acylcarnitine, and IL-6 did not change. IFN-α and IFN-γ were less than the minimal detectable concentrations (< 12.5 pg/mL and < 0.1 IU/mL, respectively) both before and after yoga.

Plasma levels of TGF-β1, BDNF, MHPG, and HVA did not change following the yoga session.

### Correlation between changes in F (or V) and changes in ANS functions and blood biomarkers

To investigate how improvement in fatigue (or vigor) relates to changes in blood biomarkers, we assessed correlations between ∆F (or ∆V) score (post F (V) value – pre F (V) value) and ∆parameter (post value – pre value of each physiological parameter and blood biomarker, shown in Table 1).

**Fig. 3:**
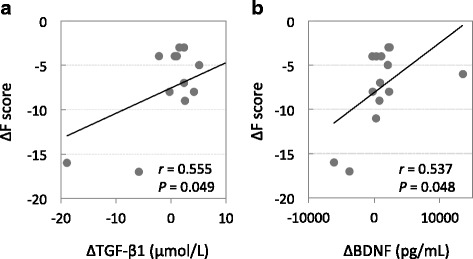
Correlations between ∆ Fatigue score and ∆ plasma TGF-β1 (**a**) and ∆ plasma BDNF (**b**). Each trend line indicates a linear relationship between two respective variables

**Fig. 4:**
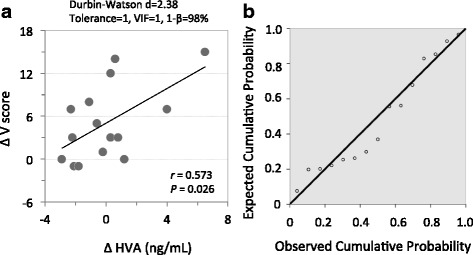
Correlations between ∆ Vigor score and ∆ plasma HVA; *n* = 15; a trendline indicates a linear relationship between two respective variables; mg/D, milligram per day (**a**). Normal P-P plot of the standardized residuals (**b**). Dependent variable: ∆ Vigor. Independent variable: ∆ HVA

Pearson’s bivariate correlation test revealed that ∆F (mean: - 8.1 ± 4.9) positively correlated with ∆TGF-β1 (mean: 1.2 ± 8.9 ng/mL, *r* = 0.555, *P* = 0.049; Fig. 3a) and ∆BDNF (mean: 1058 ± 4286.3 pg/mL, *r* = 0.537, *P* = 0.048, Fig. 3b). ∆V (mean: 5.1 ± 5.3) positively correlated with ∆HVA (mean: 0.0 ± 2.5 ng/mL, *r* = 0.573, *P* = 0.026, Fig. 4). Furthermore, multiple regression analysis showed that the increase in ∆F could not be explained by a linear increase in both ∆BDNF and ∆TGF-β1 (*r*^*2*^ = 0.314, *P* = 0.312). In contrast, 33% of the increase in ∆V was explained by a linear increase in ∆HVA (*r*^*2*^ = 0.33, *intercept* = 5.03, *B* = 1.21, *P* = 0.026, Fig. 4). There was no correlation between ∆F (or ∆V) and changes in parameters of ANS function, i.e., ∆HR, ∆HF, ∆LF, or ∆LF/HF.

## Discussion

This study demonstrated that a single session of sitting isometric yoga reduced fatigue and improved vigor in patients with CFS. Yoga also reduced HR, serum levels of cortisol and TNF-α, and had a tendency to decrease serum levels of PRL, whereas it increased the HF component of HRV and serum levels of DHEA-S. Changes in fatigue were positively correlated with changes in plasma levels of TGF-β1 and BDNF. Changes in vigor were positively correlated with changes in HVA. To our knowledge, this is the first study to investigate the effect of yoga on ANS and blood parameters in patients with CFS.

### ANS function

We found that a single session of sitting isometric yoga decreased HR and increased HF-HRV, an indicator of cardiac vagal tone, without affecting respiratory rate. Previous studies have demonstrated that, when compared with healthy subjects, patients with CFS have significantly higher resting HR [4–6], tend to have higher plasma levels of noradrenaline [4], and have significantly lower cardiac vagal indices as assessed by HRV [5, 38, 39]. That is, patients with CFS exhibit ANS alterations characterized by sympathetic overactivity and low cardiac vagal tone. Our results suggest that sitting isometric yoga improves these autonomic abnormalities. Considering the previous findings that resting HR during CFS remission was lower than during an exacerbation [40] and that reduced cardiac vagal tone was associated with cognitive impairments in patients with CFS [5], yoga-induced autonomic changes may be beneficial for patients with CFS.

### HPA axis

This study demonstrated that a single session of sitting isometric yoga reduced serum cortisol levels. This study was conducted between 2 p.m. and 4 p.m., when diurnal changes in cortisol levels are at a minimum. Therefore, decreases in cortisol may reflect the stress-relieving effects of yoga, although the possibility of diurnal decline cannot be completely ruled out. Previous studies have identified HPA axis dysfunction in patients with CFS, including attenuated diurnal cortisol rhythm and cortisol awakening response, and a cortisol surge upon awakening [7, 8]. From the current study, it is unclear if a single yoga session can improve these dysfunctions; however, regular practice of yoga has been demonstrated to recover the diurnal cortisol rhythm in cancer patients [21] and increase the cortisol awakening response in healthy subjects [41].

We also found that a single session of sitting isometric yoga increased serum DHEA-S levels. DHEA-S is a more stable sulfate derivative of DHEA, which is produced in the zona reticularis of the adrenal cortex. Several studies have demonstrated that basal levels of serum DHEA-S are lower in patients with CFS compared to healthy subjects [9–11]. DHEA-S causes promnestic effects by increasing acetylcholine release from the hippocampus [42, 43] and it buffers the negative effects of stress [44]. Considering that cognitive impairment, such as short-term memory loss, is a common symptom of CFS [1] and psychological stress exacerbates CFS symptoms [3, 25], yoga-induced decreases in cortisol levels and increases in DHEA-S levels may be beneficial in CFS patients.

### Immune function

We evaluated the effects of isometric yoga on blood levels of several cytokines, including TNF-α, IL-6, IFN-α, IFN-γ, TGF-β1, and BDNF and found that sitting isometric yoga decreased serum levels of TNF-α without affecting other cytokine levels. Several studies have suggested low-grade inflammation in patients with CFS, i.e. increased levels of several proinflammatory cytokines such as TNF-α [12, 13, 15], IL-6 [14, 15], and IFN-γ [14, 15], although these observations require confirmation [45]. One study indicated that TNF-α levels correlated with fatigue, autonomic symptoms, and flu-like malaise [13]. Therefore, sitting isometric yoga-induced decreases in TNF-α may be beneficial in patients with CFS.

This study also demonstrated that ∆F score positively correlated with ∆TGF-β1 and ∆BDNF, indicating that the fatigue-relieving effect of yoga is associated with decreased levels of these cytokines. TGF-β1 has both pro- and anti-inflammatory effects [45]. Several studies have reported that patients with CFS have higher TGF-β1 levels than healthy subjects [45, 46], which supports our finding that decreased fatigue was associated with decreased TGF-β1. In the present study, a single yoga session did not have a significant effect on the plasma levels of BDNF. Previous studies have demonstrated that regular yoga practice increases blood levels of BDNF [47, 48]. However, the role of BDNF in CFS has not yet been determined, although it is a known biomarker of depression [49–51]. Depressive disorders are a common comorbidity of CFS, suggesting the intriguing possibility that BDNF may play a role in the pathogenesis of CFS. Future studies are needed to understand the significance of BDNF in the fatigue-relieving effect of yoga in patients with CFS.

### CNS

Plasma levels of MHPG, a major metabolite of noradrenaline, and HVA, a major metabolite of dopamine, are reported to reflect noradrenergic and dopaminergic neuronal tone, respectively [52–55]. Previous studies have demonstrated that patients with CFS have significantly lower basal levels of plasma MHPG [56]. In the present study, sitting isometric yoga had no significant effect on plasma MHPG levels, suggesting that sitting isometric yoga does not affect the noradrenergic system. In contrast, sitting isometric yoga may affect the dopaminergic system. First, in this study, serum levels of PRL had a tendency to decrease after yoga. As dopamine acts as a PRL inhibiting hormone [57], this finding suggests that sitting isometric yoga may activate dopaminergic neuron activity. Second, ∆HVA positively correlated with ∆V score. This result suggests that dopaminergic activation may be related to the yoga-induced increase in vigor. Previous studies have demonstrated that patients with CFS exhibited reduced neural activation of the caudate nucleus in response to a reward task [16] and of the putamen [17], which may be due, in part, to dopaminergic dysfunction [16, 17]. Thus, reduced dopamine activity may contribute to symptoms of low energy in patients with CFS and dopaminergic activation may have a beneficial effect.

### Carnitine

Carnitine plays an important role in mitochondrial energy metabolism [58]. Several studies have suggested that CFS is associated with a reduction in plasma levels of free carnitine [59], total carnitine [59], and acylcarnitine [18, 19], whereas other studies have reported no differences between CFS patients and healthy subjects [60, 61]. The current study demonstrated that sitting isometric yoga had no effect on plasma levels of free carnitine, total carnitine, or acylcarnitine and the study failed to demonstrate any significant association between any type of carnitine and fatigue or vigor in patients with CFS.

The changes induced by sitting isometric yoga were in agreement with our original hypothesis. That is, in the short term, practicing sitting isometric yoga induced parasympathetic-dominant autonomic changes, reduced the stress hormone cortisol, increased the anti-stress hormone DHEA-S, and tended to activate the dopaminergic system within the brain of patients with CFS. Because these variables were measured just before and after yoga practice, it is uncertain how long these changes continue or whether they contribute to the long-term fatigue-relieving effect of sitting isometric yoga. According to subjective reports [2], patients described a feeling of warmth and lightness in their body and calmness and tranquility in their mind as well as reduced fatigue as some of the short-term benefits of yoga. One patient reported that these benefits lasted for one hour and gradually subsided. Therefore, it is reasonable to suggest that sitting isometric yoga-induced changes in biomarkers and ANS functions are transient. As for long-term benefits, many patients reported noticing how helpful it was to release muscular tension in their daily lives [2]. Therefore, while changes after a single yoga session may be trivial, regular yoga practice may deepen relaxation and facilitate therapeutic awareness and behavioral changes, which contribute, at least in part, to the long-term benefits of yoga.

In this study, we assessed yoga-induced changes in autonomic functions and blood biomarkers at the final yoga session with a yoga instructor, but not at the first session. There are several reasons for this. First, we wanted to know the effects of this program on patients with CFS. To achieve this goal, patients were required to be deeply familiar with the entire yoga program and able to practice it fully. Basically, the patients practiced the same 20-min program with and without a yoga instructor. In the beginning, the instructor modified the program on a patient-to-patient basis, e.g. decreasing the number of pose repetitions or skipping a certain pose, to avoid exacerbation of pain, “brain fog”, cognitive dysfunction, and post-exertional malaise. Therefore, each patient did not necessarily practice the whole program during the first practice with an instructor. Some patients reported benefits after the first session with an instructor. In contrast, some patients had difficulty memorizing the procedures in the beginning. However, these patients gradually began to feel beneficial effects, within one month of practicing sitting isometric yoga, as determined from their yoga diaries and interviews. Because of these reasons, we assessed the effects of the program at the final session only, and did not compare these results with those from the first session.

This yoga program was taught by an instructor with over 30 years of experience as a yoga instructor who had knowledge of CFS. She modified her instructions in the beginning, in consideration of each patient’s condition. As changes in several parameters, such as HF-HRV and cortisol, can be caused by psychophysiological relaxation, it is possible that the instructor’s sincere and warm attitude and personality contributed to these changes. Further studies are needed to determine if the same effect is observed with other instructors or with the DVD alone.

### Limitations

This study has several limitations. First, the sample size was relatively small, because subjects were enrolled in the yoga group in our previous randomized, controlled trial [2]. In this study, fatigue level as assessed by POMS-F score decreased remarkably (from 21.9 to 13.8) after a single session of yoga. This suggests that meaningful changes in some parameters could also be observed in this small study. On the other hand, it is possible that some meaningful changes did not reach significance due to the small sample size (e.g. serum PRL level). Further studies with larger sample sizes are necessary to determine if the decrease in PRL is significant or not. Second, blood was sampled at two time points, before and after a yoga session. These time points may provide limited information regarding the effects of sitting isometric yoga. However, considering that fatigue levels significantly decreased after just 20-min of yoga, the blood biomarkers responsible for the fatigue-relieving effect might be expected to change during this short period. Third, this study did not contain a control group. Therefore, we cannot exclude the possibility that the present findings were modulated by diurnal changes in biomarkers and ANS function or by repeated venipuncture. To address these issues, we conducted this study between 2 pm and 4 pm, when diurnal changes in parameters such as cortisol and HRV and the effect of diet on HRV were minimal. In this study, a decrease in cortisol was accompanied by an increase in DHEA-S. Even though decreases in cortisol can by caused by diurnal changes, increases in DHEA-S cannot be explained by diurnal changes because diurnal changes in DHEA-S are limited. Furthermore, even though repeated venipuncture might have had some effect on blood biomarker levels, correlations between ∆F and ∆biomarkers, e.g. ∆TGF-β1 and ∆BDNF, cannot be explained by repeated punctures. Fourth, it remains unclear if the observed changes are involved in the long-term effects of sitting isometric yoga. Therefore, we are now analyzing long-term changes in these parameters before and after the intervention period. Fifth, the study was conducted with patients who did not improve satisfactorily with conventional treatment and who could sit for at least 30 min. Further studies are required to determine if our findings can be generalized to all patients with CFS.

## Conclusions

We found that a single session of sitting isometric yoga reduced fatigue and improved vigor in patients with CFS. These changes were associated with a significant reduction in HR and serum cortisol and TNF-α levels and a marginal decrease in serum PRL, as well as a significant increase in the HF component of HRV and in serum levels of DHEA-S. Changes in fatigue were positively correlated with changes in TGF-β1 and BDNF in the blood. Vigor was positively correlated with plasma HVA. This study improves our understanding of the fatigue-relieving effect of isometric yoga in patients with CFS.

## Abbreviations

A-carn: Acylcarnitine
ANS: Autonomic nervous system
BDNF: Brain-derived neurotrophic factor
CFS: Chronic fatigue syndrome
CI: Confidence interval
CLEIA: Chemiluminescent enzyme immunoassay
CNS: Central nervous system
CV_R-R_: Coefficient of variation of R-R intervals
DHEA-S: Dehydroepiandrosterone sulfate
EASIA: Enzyme amplified sensitivity immunoassay
ELISA: Enzyme-linked immunosorbent assay
F: Fatigue
F-Carn: Free carnitine
HF: High frequency
HPA: Hypothalamic-pituitary-adrenocortical
HR: Heart rate
HRV: Heart rate variability
HVA: Homovanillic acid
IFN: Interferon
IL: Interleukin
LF: Low frequency
MHPG: 3-methoxy-4-hydroxyphenylglycol
POMS: Profile of Mood States
PRL: Prolactin
SD: Standard deviation
T-carn: Total carnitine
TGF-β1: Transforming growth factor-beta1
TNF-α: Tumor necrosis factor-α
V: Vigor

## References

[1] Fukuda K, Straus SE, Hickie I, Sharpe MC, Dobbins JG, Komaroff A (1994). The chronic fatigue syndrome: a comprehensive approach to its definition and study. International chronic fatigue syndrome study group. Ann Intern Med.

[2] Oka T, Tanahashi T, Chijiwa T, Lkhagvasuren B, Sudo N, Oka K (2014). Isometric yoga improves the fatigue and pain of patients with chronic fatigue syndrome who are resistant to conventional therapy: a randomized, controlled trial. Biopsychosoc Med.

[3] Oka T (2013). Influence of psychological stress on chronic fatigue syndrome. Adv Neuroimmune Biol.

[4] Boneva RS, Decker MJ, Maloney EM, Lin JM, Jones JF, Helgason HG, Heim CM, Rye DB, Reeves WC (2007). Higher heart rate and reduced heart rate variability persist during sleep in chronic fatigue syndrome: a population-based study. Auton Neurosci.

[5] Beaumont A, Burton AR, Lemon J, Bennett BK, Lloyd A, Vollmer-Conna U (2012). Reduced cardiac vagal modulation impacts on cognitive performance in chronic fatigue syndrome. PLoS One.

[6] De Becker P, Roeykens J, Reynders M, McGregor N, De Meirleir K (2000). Exercise capacity in chronic fatigue syndrome. Arch Intern Med.

[7] Powell DJ, Liossi C, Moss-Morris R, Schlotz W (2013). Unstimulated cortisol secretory activity in everyday life and its relationship with fatigue and chronic fatigue syndrome: a systematic review and subset meta-analysis. Psychoneuroendocrinology.

[8] Papadopoulos AS, Cleare AJ (2011). Hypothalamic-pituitary-adrenal axis dysfunction in chronic fatigue syndrome. Nat Rev Endocrinol.

[9] Kuratsune H, Yamaguti K, Sawada M, Kodate S, Machii T, Kanakura Y, Kitani T (1998). Dehydroepiandrosterone sulfate deficiency in chronic fatigue syndrome. Int J Mol Med.

[10] Scott LV, Salahuddin F, Cooney J, Svec F, Dinan TG (1999). Differences in adrenal steroid profile in chronic fatigue syndrome, in depression and in health. J Affect Disord.

[11] van Rensburg SJ, Potocnik FC, Kiss T, Hugo F, van Zijl P, Mansvelt E, Carstens ME, Theodorou P, Hurly PR, Emsley RA (2001). Serum concentrations of some metals and steroids in patients with chronic fatigue syndrome with reference to neurological and cognitive abnormalities. Brain Res Bull.

[12] Moss RB, Mercandetti A, Vojdani A (1999). TNF-alpha and chronic fatigue syndrome. J Clin Immunol.

[13] Maes M, Twisk FN, Kubera M, Ringel K (2012). Evidence for inflammation and activation of cell-mediated immunity in Myalgic encephalomyelitis/chronic fatigue syndrome (ME/CFS): increased interleukin-1, tumor necrosis factor-alpha, PMN-elastase, lysozyme and neopterin. J Affect Disord.

[14] Garcia MN, Hause AM, Walker CM, Orange JS, Hasbun R, Murray KO (2014). Evaluation of prolonged fatigue post-West Nile virus infection and association of fatigue with elevated antiviral and proinflammatory cytokines. Viral Immunol.

[15] Khaiboullina SF, DeMeirleir KL, Rawat S, Berk GS, Gaynor-Berk RS, Mijatovic T, Blatt N, Rizvanov AA, Young SG, Lombardi VC (2015). Cytokine expression provides clues to the pathophysiology of gulf war illness and myalgic encephalomyelitis. Cytokine.

[16] Miller AH, Jones JF, Drake DF, Tian H, Unger ER, Pagnoni G (2014). Decreased basal ganglia activation in subjects with chronic fatigue syndrome: association with symptoms of fatigue. PLoS One.

[17] Mizuno K, Kawatani J, Tajima K, Sasaki AT, Yoneda T, Komi M, Hirai T, Tomoda A, Joudoi T, Watanabe Y (2016). Low putamen activity associated with poor reward sensitivity in childhood chronic fatigue syndrome. Neuroimage Clin.

[18] Kuratsune H, Yamaguti K, Takahashi M, Misaki H, Tagawa S, Kitani T (1994). Acylcarnitine deficiency in chronic fatigue syndrome. Clin Infect Dis.

[19] Kuratsune H, Yamaguti K, Lindh G, Evengard B, Takahashi M, Machii T, Matsumura K, Takaishi J, Kawata S, Langstrom B (1998). Low levels of serum acylcarnitine in chronic fatigue syndrome and chronic hepatitis type C, but not seen in other diseases. Int J Mol Med.

[20] Nagendra H, Kumar V, Mukherjee S (2015). Cognitive behavior evaluation based on physiological parameters among young healthy subjects with yoga as intervention. Comput Math Methods Med.

[21] Chandwani KD, Perkins G, Nagendra HR, Raghuram NV, Spelman A, Nagarathna R, Johnson K, Fortier A, Arun B, Wei Q (2014). Randomized, controlled trial of yoga in women with breast cancer undergoing radiotherapy. J Clin Oncol.

[22] Bower JE, Greendale G, Crosswell AD, Garet D, Sternlieb B, Ganz PA, Irwin MR, Olmstead R, Arevalo J, Cole SW (2014). Yoga reduces inflammatory signaling in fatigued breast cancer survivors: a randomized controlled trial. Psychoneuroendocrinology.

[23] Rao RM, Nagendra HR, Raghuram N, Vinay C, Chandrashekara S, Gopinath KS, Srinath BS (2008). Influence of yoga on postoperative outcomes and wound healing in early operable breast cancer patients undergoing surgery. Int J Yoga.

[24] Bower JE, Garet D, Sternlieb B, Ganz PA, Irwin MR, Olmstead R, Greendale G (2012). Yoga for persistent fatigue in breast cancer survivors: a randomized controlled trial. Cancer.

[25] Oka T, Kanemitsu Y, Sudo N, Hayashi H, Oka K (2013). Psychological stress contributed to the development of low-grade fever in a patient with chronic fatigue syndrome: a case report. Biopsychosoc Med.

[26] Oka T (2015). Efficacy and limitations of autogenic training as a treatment of chronic fatigue syndrome. Jpn J Autogenic Ther.

[27] Oka T, Okumi H, Nishida S, Ito T, Morikiyo S, Kimura Y, Murakami M (2014). Effects of Kampo on functional gastrointestinal disorders. Biopsychosoc Med.

[28] Carruthers BM, van de Sande MI, De Meirleir KL, Klimas NG, Broderick G, Mitchell T, Staines D, Powles AC, Speight N, Vallings R (2011). Myalgic encephalomyelitis: international consensus criteria. J Intern Med.

[29] Institute-of-Medicine. Beyond Myalgic encephalomyelitis/chronic fatigue syndrome: Redefining an Illness. Committee on the Diagnostic Criteria for Myalgic Encephalomyelitis/Chronic Fatigue Syndrome; Board on the Health of Select Populations; Washington (DC): The National Academies Press; 2015. 25695122

[30] Matsushita T, Oka T (2015). A large-scale survey of adverse events experienced in yoga classes. Biopsychosoc Med.

[31] Oka T, Wakita H, Kimura K (2017). Development of a recumbent isometric yoga program for patients with severe chronic fatigue syndrome/myalgic encephalomyelitis: a pilot study to assess feasibility and efficacy. Biopsychosoc Med.

[32] McNair D, Lorr M, Droppleman L (1971). Mannual for the profile of mood states (POMS).

[33] Kobayashi Y, Fujikawa T, Kobayashi H, Sumida K, Suzuki S, Kagimoto M, Okuyama Y, Ehara Y, Katsumata M, Fujita M (2017). Relationship between arterial stiffness and blood pressure drop during the sit-to-stand test in patients with diabetes mellitus. J Atheroscler Thromb.

[34] Sawada Y, Ohtomo N, Tanaka Y, Tanaka G, Yamakoshi K, Terachi S, Shimamoto K, Nakagawa M, Satoh S, Kuroda S (1997). New technique for time series analysis combining the maximum entropy method and non-linear least squares method: its value in heart rate variability analysis. Med Biol Eng Comput.

[35] Jia T, Ogawa Y, Miura M, Ito O, Kohzuki M (2016). Music attenuated a decrease in parasympathetic nervous system activity after exercise. PLoS One.

[36] Shaffer F, McCraty R, Zerr CL (2014). A healthy heart is not a metronome: an integrative review of the heart's anatomy and heart rate variability. Front Psychol.

[37] Legey S, Lamego MK, Lattari E, Campos C, Paes F, Sancassiani F, Mura G, Carta MG, Rocha NB, Nardi AE (2016). Relationship among body image, anthropometric parameters and mental health in physical education students. Clin Pract Epidemiol Ment Health.

[38] Sisto SA, Tapp W, Drastal S, Bergen M, DeMasi I, Cordero D, Natelson B (1995). Vagal tone is reduced during paced breathing in patients with the chronic fatigue syndrome. Clin Auton Res.

[39] Yamagiti K, Tajima S, Kuratsune H (2013). Autonomic dysfunction in chronic fatigue syndrome. Adv Neuroimmune Biol.

[40] Miwa K, Fujita M (2009). Cardiac function fluctuates during exacerbation and remission in young adults with chronic fatigue syndrome and "small heart". J Cardiol.

[41] Cahn BR, Goodman MS, Peterson CT, Maturi R, Mills PJ (2017). Yoga, meditation and mind-body health: increased BDNF, cortisol awakening response, and altered inflammatory marker expression after a 3-month yoga and meditation retreat. Front Hum Neurosci.

[42] Roberts E, Bologa L, Flood JF, Smith GE (1987). Effects of dehydroepiandrosterone and its sulfate on brain tissue in culture and on memory in mice. Brain Res.

[43] Wolkowitz OM, Reus VI, Roberts E, Manfredi F, Chan T, Raum WJ, Ormiston S, Johnson R, Canick J, Brizendine L (1997). Dehydroepiandrosterone (DHEA) treatment of depression. Biol Psychiatry.

[44] Morgan CA, Southwick S, Hazlett G, Rasmusson A, Hoyt G, Zimolo Z, Charney D (2004). Relationships among plasma dehydroepiandrosterone sulfate and cortisol levels, symptoms of dissociation, and objective performance in humans exposed to acute stress. Arch Gen Psychiatry.

[45] Blundell S, Ray KK, Buckland M, White PD (2015). Chronic fatigue syndrome and circulating cytokines: a systematic review. Brain Behav Immun.

[46] Kennedy G, Spence V, Underwood C, Belch JJ (2004). Increased neutrophil apoptosis in chronic fatigue syndrome. J Clin Pathol.

[47] Pal R, Singh SN, Chatterjee A, Saha M (2014). Age-related changes in cardiovascular system, autonomic functions, and levels of BDNF of healthy active males: role of yogic practice. Age (Dordr).

[48] Lee M, Moon W, Kim J (2014). Effect of yoga on pain, brain-derived neurotrophic factor, and serotonin in premenopausal women with chronic low back pain. Evid Based Complement Alternat Med.

[49] Shimizu E, Hashimoto K, Okamura N, Koike K, Komatsu N, Kumakiri C, Nakazato M, Watanabe H, Shinoda N, Okada S (2003). Alterations of serum levels of brain-derived neurotrophic factor (BDNF) in depressed patients with or without antidepressants. Biol Psychiatry.

[50] Aydemir O, Deveci A, Taneli F (2005). The effect of chronic antidepressant treatment on serum brain-derived neurotrophic factor levels in depressed patients: a preliminary study. Prog Neuro-Psychopharmacol Biol Psychiatry.

[51] Lee BH, Kim H, Park SH, Kim YK (2007). Decreased plasma BDNF level in depressive patients. J Affect Disord.

[52] Kopin IJ (1985). Catecholamine metabolism: basic aspects and clinical significance. Pharmacol Rev.

[53] Nagaoka S, Iwamoto N, Arai H (1997). First-episode neuroleptic-free schizophrenics: concentrations of monoamines and their metabolites in plasma and their correlations with clinical responses to haloperidol treatment. Biol Psychiatry.

[54] Yoshimura R, Nakamura J, Shinkai K, Ueda N (2004). Clinical response to antidepressant treatment and 3-methoxy-4-hydroxyphenylglycol levels: mini review. Prog Neuro-Psychopharmacol Biol Psychiatry.

[55] Umene-Nakano W, Yoshimura R, Ueda N, Suzuki A, Ikenouchi-Sugita A, Hori H, Otani K, Nakamura J (2010). Predictive factors for responding to sertraline treatment: views from plasma catecholamine metabolites and serotonin transporter polymorphism. J Psychopharmacol.

[56] Demitrack MA, Gold PW, Dale JK, Krahn DD, Kling MA, Straus SE (1992). Plasma and cerebrospinal fluid monoamine metabolism in patients with chronic fatigue syndrome: preliminary findings. Biol Psychiatry.

[57] Ben-Jonathan N, Hnasko R (2001). Dopamine as a prolactin (PRL) inhibitor. Endocr Rev.

[58] Rebouche CJ, Engel AG (1983). Carnitine metabolism and deficiency syndromes. Mayo Clin Proc.

[59] Majeed T, de Simone C, Famularo G, Marcellini S, Behan PO (1995). Abnormalities of carnitine metabolism in chronic fatigue syndrome. Eur J Neurol.

[60] Plioplys AV, Plioplys S (1995). Serum levels of carnitine in chronic fatigue syndrome: clinical correlates. Neuropsychobiology.

[61] Jones MG, Goodwin CS, Amjad S, Chalmers RA (2005). Plasma and urinary carnitine and acylcarnitines in chronic fatigue syndrome. Clin Chim Acta.

